# Stage Children

**Published:** 1911-03-25

**Authors:** 


### Stage Children.

If one suggests that it is not desirable for young children to appear on the stage, one is supposed to cherish a puritanic dislike to the theatre and all connected with it. The conclusion does not necessarily follow. One might as well say that the objection to young children working in factories implies a hatred of the textile trades. But a place which is a lawful and even desirable sphere of work for an adult may be a very bad place for a child, and for other than moral reasons, though these are not to be ignored. Some people, indeed, draw a distinction, and say that while it is not desirable for a child to take part in what is called a "problem play," it can do him or her—it is generally her—no harm to play in a pantomime or in such a child's play as "The Blue Bird" or "Pinkie and the Fairies." But although these two plays are particularly charming examples of the drama which turns on the ideas and fancies of childhood, it does not follow that it is a good thing for children to take part in them. In the first place, the child-performers are kept up to hours which few of us would like to see emulated by our own children. Secondly, these clever performances do not entirely "come by Nature," however much natural talent they may indicate. The dainty dancing of the scores of little girls whom one may see in our theatres means months, if not years, of training. They may enjoy the training (as a rule they do), but meanwhile the chances are that their ordinary education is suffering, and that in adult life they may feel the want of it. It is true that in some schools lessons on ordinary subjects are given, but after arduous practice at work which takes so much out of both brain and nerve as acting, singing, or dancing, it may be doubted if the pupils profit by the teaching afterwards given more than does the ordinary half-timer, whom most people wish to see done away with. Again, the work on the stage involves a nervous strain which is unwholesome for children, and especially for girl-children. At the time when they should be accumulating strength of every kind for the work of adult life, they are, unconsciously and for the benefit of others, spending their nerve-capital in the most reckless way. It is not necessary to dwell on the jealousies and rivalries that the work fosters, though no wise parent will forget the evil effect of this. And though rivalry and emulation exist in ordinary schools, they are not so acute as in this sphere, where public applause is the nightly reward of success, and where failure in the lighter arts cannot be made up for by success in more serious studies. Finally, one must remember that we call a child's performance clever simply because it is the performance of a child. Done by an adult it might fail to win our admiration, and unless these children go on improving to a point which implies genius—and genius is rare—they may find themselves on a lower rung of the ladder of success in mature life than they were at the beginning of it. Nay, they may see themselves passed by some who have less experience than they, but whose nerves and voices have not been overtaxed in their childhood.

